# Do AKT1, COMT and FAAH influence reports of acute cannabis intoxication experiences in patients with first episode psychosis, controls and young adult cannabis users?

**DOI:** 10.1038/s41398-020-0823-9

**Published:** 2020-05-12

**Authors:** Chandni Hindocha, Diego Quattrone, Tom P. Freeman, Robin M. Murray, Valeria Mondelli, Gerome Breen, Charles Curtis, Celia J. A. Morgan, H. Valerie Curran, Marta Di Forti

**Affiliations:** 1 Clinical Psychopharmacology Unit, Research Department of Clinical, Educational and Health Psychology, University College London, London, United Kingdom; 2 Translational Psychiatry Research Group, Research Department of Mental Health Neuroscience, Division of Psychiatry, Faculty of Brain Sciences, University College London, London, United Kingdom; 3 NIHR University College London Hospitals Biomedical Research Centre, University College Hospital, London, United Kingdom; 4 Social, Genetic and Developmental Psychiatry Centre, Institute of Psychiatry, Psychology and Neuroscience, King’s College London, London, SE5 8AF UK; 5 National Institute for Health Research (NIHR) Maudsley Biomedical Research Centre at South London and Maudsley NHS Foundation Trust and King’s College London, London, UK; 6 South London and Maudsley NHS Mental Health Foundation Trust, London, UK; 7 Addiction and Mental Health Group (AIM), Department of Psychology, University of Bath, Bath, UK; 8 NIHR BioResource Centre Maudsley, NIHR Maudsley Biomedical Research Centre (BRC) at South London and Maudsley NHS Foundation Trust (SLaM), London, UK; 9 Department of Psychological Medicine, Institute of Psychiatry, Kings College London, De Crespigny Park, SE5 8AF London, UK; 10 Department of Psychosis Studies, Institute of Psychiatry, King’s College London, De Crespigny Park, Denmark Hill, London, SE5 8AF UK; 11 Psychopharmacology and Addiction Research Centre (PARC), University of Exeter, Exeter, UK

**Keywords:** Clinical genetics, Schizophrenia

## Abstract

Epidemiological and biological evidence support the association between heavy cannabis use and psychosis. However, it is unclear which cannabis users are susceptible to its psychotogenic effect. Therefore, understanding genetic factors contributing to this relationship might prove an important strategy to identify the mechanisms underlying cannabis-associated psychotic experiences. We aimed to determine how variation in AKT1, COMT and FAAH genotypes, and their interaction with three different groups (first episode psychosis (FEP) patients (*n* = 143), controls (*n* = 92) and young adult (YA) cannabis users *n* = 485)) influenced cannabis experiences, in those who had used cannabis at least once. We investigated the role of AKT1 (rs2494732), COMT Val158Met (rs4680) and FAAH (rs324420) on cannabis experiences by combining data from a large case-control study of FEP patients, with a naturalistic study of YA cannabis users (*n* = 720). Outcome measures were cannabis-induced psychotic-like experiences (cPLEs) and euphoric experiences (cEEs). We used linear mixed effects models to assess the effects of each genotype and their interaction with group, adjusting for age, sex, ethnicity, age of first cannabis use, years of use and frequency. cPLEs were more frequent in FEP patients than controls and YA cannabis users. cEEs were more prevalent in YA cannabis users than FEP patients or controls. Variation in AKT1, COMT or FAAH was not associated with cPLEs/cEEs. There was no interaction between genotype and group (FEP cases, controls and YA cannabis users) on cPLEs/cEEs. In conclusion, AKT1, COMT or FAAH did not modulate specific psychotomimetic response to cannabis and did not interact with group, contrary to previous research.

## Introduction

The use of cannabis has been associated with a 3.9-fold increase in the risk of schizophrenia and other psychosis-related outcomes among the heaviest cannabis users compared to the nonusers^1–3^, but only a small minority who use the drug will develop psychotic symptomology. Although there is a debate about causality in the relationship between cannabis and psychosis^4,5^, there is now new evidence that show that daily use of high potency cannabis types contributes to rates of psychotic disorders. In cities like Amsterdam and London, 50% and 30% of new cases of psychotic disorders can be attributed to the use of high potency cannabis, respectively^6^. Indeed several studies have shown that cannabis does produce transient psychotomimetic effects, which are common and experienced by nonclinical populations^7^. The term psychotomimetic implies mimicry of a wide range of experiences observed in the psychosis continuum; and these may include paranoia, hallucinations or euphoria^8^. These cannabis-induced experiences may be a marker of a vulnerability to psychosis, with those being most vulnerable, experiencing the greatest psychotomimetic effects^9–12^. However, there is marked interindividual variability in this cannabis response. As well as environmental factors such as pattern of cannabis use, genetic factors are thought to play a key role in the differences in cannabis sensitivity to the drug’s psychotomimetic effects.

Genetic differences in the dopaminergic system may interact with cannabis use to increase the risk for the development of psychosis^13,14^. Some evidence suggests that delta-9-tetrahydrocannabinol (THC), the primary psychoactive component in cannabis, acutely increases dopamine release in the human striatum^15–18^, but studies have been small and inconsistent^19^. The catechol-o-methyltransferase (COMT) gene encodes an enzyme that breaks down catecholamines such as dopamine. COMT is specifically important for the dopaminergic tone in the prefrontal cortex. The COMT rs4680 G/A polymorphism causes a non-synonymous change from a valine (Val) amino acid to a methionine (Met) amino acid. This amino-acid change leads to a three-to-fourfold reduction in COMT activity, and therefore greater levels of dopamine in the prefrontal cortex^20,21^. Caspi et al.^13^ found a gene × environment interaction between allelic variation in COMT and cannabis use on the development of schizophrenia wherein Val alleles were identified as the risk allele for psychosis (*n* = 953)^13^. It follows that those with the risk allele (Val) would show greater psychotomimetic effects in response to cannabis as found by Henquet et al.^22–25^. However, subsequent findings have been shown to be inconsistent and rs4680 has not been identified as a causal variant in the latest schizophrenia Genome Wide Association Studies (GWAS)^26–30^. One recent review suggests this inconsistency may be a result of study design in that the interaction was only significant in case-only studies but not in studies that used other clinical outcomes/nonclinical psychosis and that future studies should utilise additional control groups^31^.

The Protein Kinase B family, which consists of three serine/threonine kinases (AKT1, 2 and 3), is another integral component of dopaminergic signalling. It acts downstream of the dopamine D2 receptor. The AKT1 gene has been associated with schizophrenia in several independent samples^32–34^, although these findings are not consistently replicated^35,36^. A replicable gene × environment interaction in which the minor C allele of the rs2494732 SNP has been associated with an increased likelihood of developing a psychotic disorder in those with a history of cannabis use (*n* = 489-801 patients and 278–704 controls/unaffected siblings, respectively)^37,38^. Among daily users, this increased seven-fold for C carriers in comparison to TT carriers^37^. Recent research suggests that genetic variation in AKT1 is involved in the cognitive effects of cannabis on psychosis (ref. ^39^ *n* = 611) and on the acute psychotomimetic effects of smoked cannabis (ref. ^29^ *n* = 442) as assessed by the Psychotomimetic States Inventory (PSI; ref. ^8^). However, the functional consequences of this intronic SNP are unclear, and so far, it has not been associated with any protein change. However, data from HapMap 3^40^ has shown that rs2494732 is 702 base-pairs away from rs1130233, a SNP which does affect AKT gene messenger RNA expression^41^. Rs2494732 and rs1130233 are therefore most likely in linkage disequilibrium (r^2^ = .95), and this may explain the convergent evidence of research investigating the AKT1-cannabis interaction on both psychosis outcomes^38,42^ and altered cognitive performance^39^ when investigating both SNPs^37^. However, it should be noted that rs2494732 has not yet been identified in GWAS as a causal variant^30^.

Since cannabis primarily acts on the endocannabinoid (eCB) system, it seems pertinent to also investigate interactions within this system. The eCB system is a signalling system made up of receptors (CB1, CB2), internal ligands (anandamide and 2-AG: 2-Arachidonoylglycerol), and enzymes (Fatty Acid Amide Hydrolase (FAAH) and Monoacylglycerol lipase (MAGL)), which are responsible for degradation and reuptake of eCBs. The “cannabinoid” hypothesis of schizophrenia hypothesises that eCB over-activity may contribute to the pathophysiology of schizophrenia. Recently, meta-analysis has shown that the eCB system may be dysregulated in patients at all stages of the psychosis continuum, in comparison to controls^43^. Patients have higher levels of anandamide in their cerebrospinal fluid, blood and greater CB1 receptor expression on peripheral immune cells^43^. Bioque et al.^44^ found that first episode psychosis (FEP) patients (*n* = 95) in comparison to healthy controls (*n* = 90) had a dysregulated eCB system where they not only showed increases in the synthesising enzymes but also significant decreases in the degrading enzymes. This effect was exaggerated in FEP patients who used cannabis^44^. Anandamide levels were also found to be downregulated in heavy cannabis users without a psychotic disorder^45^ and the extent of this anandamide change was associated with a lower risk of psychotic symptoms when drug free (*n* = 20)^45^. Additionally, in a clinical trial with individual’s diagnosed with schizophrenia, cannabidiol (CBD), the non-intoxicating cannabinoid in cannabis, and potential FAAH inhibitor, increased plasma levels of anandamide and this correlated with clinical improvement (*n* = 39)^46^. It was therefore hypothesised that one of the mechanisms of clinical improvement was through inhibition of the FAAH enzyme^46^.

The FAAH rs324420 C to A polymorphism leads to a non-synonymous exchange from proline to threonine at amino acid 129 of the protein. Those who are homozygous for the A allele have a 30% reduction in the functioning of the enzyme^47–49^. The A allele can therefore act as a human genetic model of FAAH inhibition associated with increased anandamide levels, and has been shown to protective against anxiety, stress and fear-related behaviour^50,51^. Therefore, on the one hand, anandamide is reliably increased in psychotic patients^43^ and on the other, the gene associated with inhibition of FAAH, that breaks down anandamide, may be protective against anxiety. The FAAH genotype has not been investigated in relation to cannabis-induced psychotic-like effects before. However, it has been investigated in its association with population-level schizophrenia (*n* = 260) where no relationship was found^52^. Moreover, the FAAH rs324420 SNP has also not been associated with diagnosis in the latest schizophrenia GWAS^30^. Postmortem studies have also reported that enzymes that regulate endocannabinoid levels do not differ between schizophrenia and age- and sex-matched comparison participants^53^. Therefore, how the FAAH genotype and anandamide play a role in psychosis is still being debated.

In this study, we combine data from two large-scale studies. The GAP study is a case-control study based in South London^54^. The second is a naturalistic study of young adult (YA) cannabis users conducted in the UK^55^. Sections of this data have previously been published. Di Forti et al.^37^ analysed AKT1 in relation to risk of psychosis in cannabis users in the FEP sample. The AKT1 and COMT genotype data were analysed in relation to acute psychotomimetic effects in Morgan et al.^29^ in the YA study. However, for this first time, in this secondary analysis we assess participants who used cannabis who were administered a modified cannabis experiences questionnaire (CEQ), that was a priori coordinated across the two studies and here we look at the contribution of the three SNPs: AKT1 COMT and FAAH, which have not previously been analysed together in either sample. The aim of coordinating over studies was to increase the sample size, as previous candidate gene studies have been limited by their small sample sizes. We aimed to investigate between group differences (controls, YA cannabis population, and FEP patients). We hypothesised interactions between genotype and group wherein the risk allele for psychosis for each SNP will show the greatest effect on cannabis-induced psychotic-like experiences in the psychosis population, in comparison to both the case-matched controls and the YA cannabis users. For the first time, we investigate the relationship between genetic variation in the FAAH gene and its relationship to cannabis-induced experiences.

## Materials and methods

### Study participants

#### GAP study

This study utilized a subsample of the Genetic and Psychosis (GAP) study^56^. The GAP study recruited 410 patients with FEP and 370 population control participants, referred to as “GAP controls”^56^. This case-control study approached all FEP patients aged 18–64 in the Lambeth, Southwark, Croydon adult inpatient units of the South London and the Maudsley NHS trust between Dec 2005 and Oct 2010. Controls were matched on education and employment status but not cannabis use. Inclusion criteria for cases were: 18–65 years/old presenting to psychiatric services for the first time with a psychotic disorder (codes F20–29 and F30–33 from the International Classification of Diseases [ICD-10]), and resident within tightly defined catchment areas in Southeast London, UK. Exclusion criteria were: organic psychosis; intelligence quotient (IQ) under 70; previous contact with services for psychosis, and transient psychotic symptoms resulting from acute drug intoxication. Further details can be found in refs. ^37,56^. Controls were aged 18–65 years and recruited from the population locally living in the above areas and were recruited via internet and newspaper advertisements, and leaflet distribution within the local area. The Psychosis Screening Questionnaire (PSQ) was administered to all potential control group participants; individuals were excluded if they met criteria for a psychotic disorder. Participants for this analysis were included if they had completed the CEQ and therefore had used cannabis at least once, and had data available on the three SNPs, therefore the number of cases and controls from the GAP study that are analysed vary. All participants provided written, informed consent. Ethical approval was provided by the South London and Maudsley and Institute of Psychiatry Local Research Ethics Committee.

#### YA cannabis user study

The second sample was a naturalistic study of 16–24-year-old cannabis users who did not have any diagnosed psychiatric health problems (*n* = 611)^29,55,57^. In order to represent a wide range of cannabis exposure, recruitment was targeted at both recreational (1–24 days/month) and daily (≥25 days/month) users. Participants were identified through word of mouth and snowball sampling, starting with undergraduate students and the local community around UCL (Central London) between November 2008 and January 2011. Participants completed the CEQ and provided saliva samples for DNA when non-intoxicated. All participants provided written, informed consent. The study was approved by the UCL Ethics Committee and its aims were supported by the UK Home Office. To be included in the study, participants were required to speak English fluently, have no learning disability, no personal or first degree relative history of psychotic illness and have normal or corrected to-normal vision.

### 2.2 Assessments

#### Cannabis experiences questionnaire (CEQ)

Modified from Barkus et al.^10^ and utilised in Di Forti et al.^56^, this questionnaire assesses the lifetime frequency of nine intoxication experiences, six are psychotic-like experiences (cPLE; feeling fearful; feeling crazy or mad; feeling nervy; feeling suspicious; hearing voices; seeing visions), and three are euphoric experiences (cEE; feeling happy; understanding the world better; being full of plans or ideas). They were rated on a 5 point Likert scale: (0 rarely or never, 1 from time to time, 2 sometimes, 3 more often than not, 4 almost always). Factor analysis of the CEQ suggests that individual items load onto two scales—cPLE and cEE^58^ and these were used as our two outcome variables.

#### Demographics and cannabis use

In both studies, whilst non-intoxicated, participants provided demographic details including Age, Sex (male/female) and Ethnicity (White British, White Other, Mixed, Indian, Pakistani, Bangladeshi, Other Asian, black Caribbean, Black African, Black other, Chinese, Other). Cannabis use history variables included age of first cannabis use (years), number of years of cannabis use and frequency of cannabis use (categories: everyday; more than once a week; a few times a month; a few times each year; only once or twice).

### Genotyping

In both studies, DNA extraction was performed using standard phenol–chloroform methods for all samples. Off the shelf Taqman assays for these polymorphisms are available as a kit (Applied Biosystems, Life Technologies, Paisley, UK https://www.thermofisher.com/uk/en/home/brands/applied-biosystems.html). In the GAP study, 75% samples were blood and 25% were cheek swabs^37^. A comparison of genotype results for 360 individuals with overlapping blood and cheek swab DNA revealed there was 100% concordance between blood- and cheek-derived genotype data. In the cannabis user study, 100% of DNA was obtained by cheek swabs.

As the purpose of this study was to explicitly test for an interaction at three SNPs, we focused on AKT1: (rs2494732), COMT: Val158Met (rs4680), and FAAH (rs324420). Genotype calls were discriminated based on algorithmic membership of three clusters representing TT/CT/CC genotype classes for AKT1 rs2494732. AA/AG/GG genotype classes for COMT rs4680, and CC/AC/AA genotype classes for FAAH rs324420. The nomenclature was based on previous research^29,31,59^. In order to increase the power to detect an interaction, for AKT1, those with the minor allele CC were combined with heterozygotes CT. For COMT, GG and AG were combined and for FAAH, the minor allele A was combined with the heterozygote AC.

### Statistical analysis

Statistical analysis was conducted using SPSS version 24; IBM, Chicago, IL, USA and Stata/IC v. 15.1 (StataCorp, College Station, TX). The code is available from the corresponding author at request. An a priori power calculation conducted with G*Power to calculate the sample size required to achieve a study power of 80% at a 5% significance level and effect size of f = 0.1 suggested 81 participants were required; with 10 predictors. A small effect size was chosen in line with previous behavioural genetics research.

Participants were included in the statistical analysis if they had used cannabis at least once, completed the CEQ and had genetic data available. We calculated cPLEs and cEEs by simple summation as per Sami et al.^60,61^. There were half as many euphoric items as psychotic-like items, so scores for euphoric items were doubled. Where a single item was missing, we imputed the mean of the subscale into the item, and recalculated the subscale. This was the case for seven participant’s cPLE scores and four participant’s cEE scores. If more than one item was missing for the subscale and precluded the calculation of a subscale, this was considered missing data (1.25% of data).

We compared the three groups on demographics, cannabis use variables and genotypes using Chi-squared tests and one-way analysis of variance (ANOVA). Extreme values >3x Standard Deviation were winsorized to the next highest value +1 SD (non-outlier). This was the case for <1% of the continuous data. When homogeneity of variance could not be assumed, we conducted a Brown Forsythe test. Pairwise comparisons were Bonferroni corrected.

We conducted linear mixed effects models (LMMs) regressing the genotypes, group and their interaction onto the two outcome variables (cPLE and cEE) in individual models. Only full cases were analysed. In every model, we co-varied for the other subscale of the CEQ following Sami et al.^60,61^, and because there was a small but significant correlation between cPLEs and cEEs (*r*(719)=0.13, *p* = 0.001). Sex was coded as 0 (male) or 1 (female), with males being the reference category. Self-reported ethnicity was coded by category. The YA cannabis user study did not have ethnicity derived from genetic data. To confirm self-report of ethnicity in the GAP sample, genetic ancestry was derived using a panel of 57 ancestry informative genetic markers^37^. These were genotyped using iPLEX technology developed for the MassArray platform (Sequenom Inc., San Diego, California). Eighty-three percent of participants had information on both self-reported ethnicity and ancestry markers in the GAP study. The level of overall agreement between self-reported and genetic ethnicities (96%) was reassuringly high in the GAP study. In this analysis, we correlated the genetic and self-reported ethnicity in the GAP study, which showed a strong correlation (*R*(32)=0.7, *p* < 0.01); so we utilised self-reported ethnicity for both studies. Frequency of cannabis use was coded from 0–4, with “everyday” being the reference category such that a negative beta means greater frequency of cannabis use associated with the cannabis-induced experience. Group was coded to infer increasing risk of psychosis with 0 = controls from the GAP study, 1 = YA cannabis users and 2 = FEP patients from the GAP study. AKT1 was coded as 0 (TT) or 1 (CC or CT). COMT was coded as (0- AA i.e. MET/MET) or 1 (GG or AG i.e. VAL/VAL or VAL/MET). FAAH was coded as 0 (CC) or 1 (AA or AC). The gene × group interaction was therefore calculated as SNP (0, 1) × group (0, 1, 2), with the reference categories being GAP controls with the lowest genetic risk. The final LMMs were checked for violation of assumptions. All final models include a random intercept for ‘participant’ to account for additional residual variability. Unadjusted associations from linear regression between each variable with cPLEs and cEEs can be found in Supplementary materials 1. The unstructured variance-covariance structure was selected. Multicolinearity was not an issue (all VIFs >1 & <5). All *P* values were thresholded at *P* < .05 (FDR-corrected for multiple comparisons). All main effects and interactions were compared against this alpha. Finally, given differences in ethnic groups across allele frequencies, we conducted sensitivity analyses without black individuals (supplementary materials Tables 1–3 sensitivity analysis 1) and also within just the major ethnicity group i.e. white European (supplementary materials Table 4–6 sensitivity analysis 2).

## Results

### Demographics and cannabis use variables

Data were available for a total of 720 participants all of whom had used cannabis at least once and completed the CEQ (Table 1). As per Table 1, groups varied significantly on demographic and drug use variables. YA cannabis users were younger than FEP patients (*p* < 0.001) and GAP controls (*p* < 0.001). No difference in age emerged between FEP patients and GAP controls (*p* = 0.056). The GAP controls had a more even distribution of males to females than the FEP patients and the YA cannabis users (*χ*^*2*^(2) = 6.5 *p* = 0.038). The YA cannabis users had more white British individuals and the GAP study (FEP and controls) had more black Caribbean, African and black other participants, likely due to differences in sampling strategy. In regards to cannabis use, the YA cannabis users started at an earlier age than the FEP patients (*p* < 0.001) and GAP controls (*p* < 0.001). No difference emerged between FEP patients and GAP controls in the GAP study (*p* = 1.00). YA cannabis users had smoked cannabis for less years than FEP patients (*p* < 0.001) and GAP controls (*p* < 0.001). Cannabis frequency significantly differed between groups (χ^2^(10) = 99.1, *p* ≤ 0.001). FEP patients had relatively more individuals using cannabis everyday followed by the YA cannabis users and the GAP controls (Supplementary Fig. 1). There was a similar number of daily and ‘more than weekly’ users in the YA cannabis users, and there were more GAP controls who used cannabis ‘more than once a week’ in comparison to FEP patients (Supplementary Fig. 1).

**Table 1:** Group data for demographics, cannabis use data and genetics.

	Whole sample (*n* = 720)	FEP patients (GAP) (*n* = 143)	GAP controls (*n* = 92)	YA cannabis users (485)	Test statistic
Age at testing (M(SD))	23.5 (6.6)	28.4 (8.36)	30.1 (9.5)	20.7 (1.8)	*F*(2,204.6) = 82.2, *p* ≤ 0.001, ɳ^2^ = 0.36^a^
Gender (N)	M: 489 F: 231	M: 97 F: 46	M: 52 F: 40	M: 340 F: 145	*χ*^*2*^(2) = 6.5, *p* = 0.038, V = 0.10
Ethnicity (N)					*χ*^*2*^(22) = 158.424, *p* ≤ 0.001, V = 0.30
White British	359	49	44	266	
White Other	85	16	17	52	
Mixed	45	14	4	27	
Indian	38	0	2	36	
Pakistani	8	0	1	7	
Bangladeshi	5	4	0	1	
Other Asian	48	5	2	41	
Black Caribbean	50	26	15	9	
Black African	36	20	6	10	
Black other	3	3	0	0	
Chinese	17	1	0	16	
Other	25	5	1	19	
Age of first cannabis use (M(SD))	15.3 (3.0)	16.3 (5.0)	16.3 (3.0)	14.9 (2.0)	*F*(2,166.53) = 10.04, *p* ≤ 0.001, ɳ^2^ = 0.04^a^
Years of cannabis use (M(SD))	6.34 (5.3)	10.06	9.95 (8.9)	4.8 (2.5)	*F*(2,155) = 31.8, *p* ≤ 0.001, ɳ^2^ = 0.19^a^
AKT1 (N)	635	102	70	436	*χ*^2^(4) = 2.27, *p* > 0.05, V = 0.04
TT	160 (25.2%)	22 (21.6%)	17 (24.3%)	121 (26.1%)	
CT	326 (51.3%)	54 (52.9%)	40 (57.1%)	232 (50.1%)	
CC	149 (23.5%)	26 (25.5%)	13 (18.6%)	110 (23.8%)	
*HW stat*	*χ2* = 1.17, *p* > 0.05	*χ*^*2*^ = 0.37, *p* > 0.05	*χ*^*2*^ = 1.5, *p* > 0.05	*χ*^*2*^ = 0.0 *p* > 0.05	
COMT (N)	657	113	91	453	*χ*^*2*^(4) = 16.93, *p* = 0.002, V = 0.11
AA	143 (21.8%)	11 (9.7%)	15 (16.5%)	117 (25.8%)	
AG	316 (48.1%)	58 (51.3%)	46 (50.5%)	212 (46.8%)	
GG	198 (30.1%)	44 (38.9%)	30 (33%)	124 (27.4%)	
*HW stat*	*χ*^*2*^ = 0.64 *p* > 0.05	*χ*^*2*^ = 0.9, *p* > 0.05	*χ*^*2*^ = 0.14, *p* > 0.05	*χ*^*2*^ = 1.8, *p* > 0.05	
FAAH (N)	654	115	84	455	χ^2^(4) = 12.24, *p* = 0.016, V = 0.10
CC	409 (62.5%)	71 (61.7%)	42 (50%)	296 (65.1%)	
AC	206 (31.5%)	39 (33.9%)	21 (36.9%)	136 (29.9%)	
AA	39 (6.0%)	5 (4.3%)	11 (13.1%)	23 (5.1%)	
Cannabis-induced –Psychotic-Like experiences (M(SD))	9.26 (3.68)	10.54 (4.74)	8.90 (3.16)	9.00 (3.33)	F(2,288.58) = 9.43, *p* ≤ 0.001, ɳ^2^ = 0.02^a^
Cannabis- induced Euphoric Experiences (M(SD))	17.86 (5.97)	16.91 (7.20)	*15.28 (5.96)*	M = 18.6 (5.38)	F(2, 293.85) = 12.57 *p* < 0.001, ɳ^2^ = 0.04^a^

*HW* Hardy Weinberg, *ɳ*^*2*^ eta squared, *V* Cramer’s V.

^a^Brown-Forsyth Test.

### Group differences in cannabis experiences

**Fig. 1:**
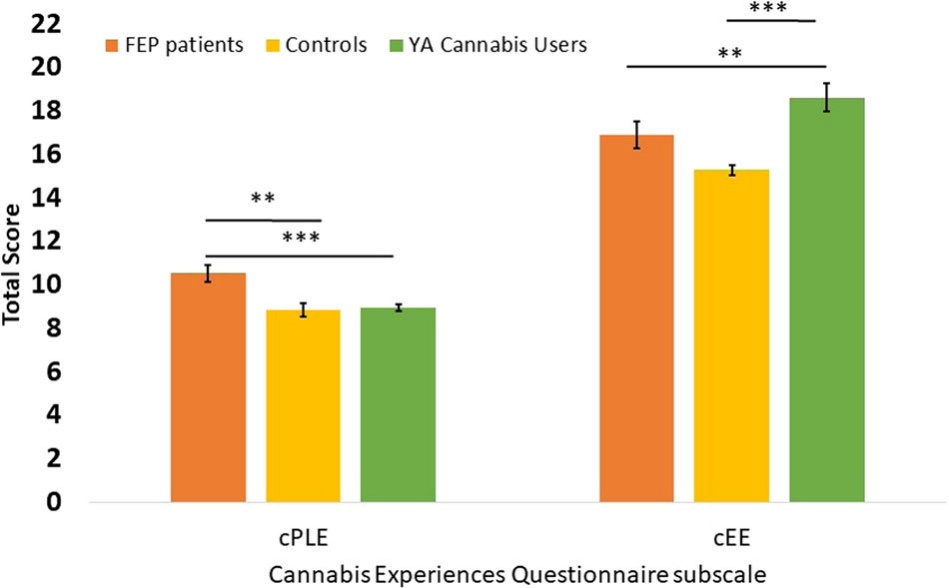
Cannabis-induced expereinces by group. Mean (±SEM) scores for cannabis-induced psychotic-like experiences (cPLE) and euphoric experiences (cEE) based on group (FEP patients, GAP controls and YA cannabis users). Bonferroni corrected pairwise comparisons are shown. Overall psychotic cases experienced most psychotic symptoms but cannabis users experienced most euphoric symptoms. ***p < 0.001, **p < 0.01 *p < 0.05.

Score on cPLEs and cEEs, are shown in Fig. 1 and Table 1. There were significant differences between the three groups on cPLEs (*F*(2,708) = 10.85, *p* < 0.001). There was little difference between the GAP controls and the YA cannabis users but both differed significantly from the FEP patients who experienced greater cPLEs (*p*’s ≤ 0.002). In regards to cEEs, there were significant differences between the three groups (*F*(2,716) = 14.99, *p* < 0.001). The YA cannabis users experienced greater cEEs than GAP controls *(p* < 0.001) and FEP patients (*p* = 0.006). There was no difference between the FEP patients and the GAP controls (*p* = 0.11). Given that YA cannabis users were younger than both FEP patients and GAP controls and therefore had less years of cannabis use, the effects on cPLEs and cEEs may be a function of age. Therefore as an additional check, we restricted the analysis to those who were between 16–24 years old and replicated the group differences.

### Genotypic frequencies

Across all participants, genotypes were in Hardy Weinberg Equilibrium (HWE). HWE was violated for the distribution of FAAH in GAP control sample only; however, this did not modify the overall HWE for FAAH (Table 1).

### Linear mixed effects models

#### AKT1

In the model predicting cPLEs, there was a positive association between cEEs and cPLEs (B:0.09, 95% CI:0.04 to 0.14) (Table 2). There was negative association with age such that younger individuals had greater cPLEs (B: −0.13, 95% CI:−0.25 to −0.02) but this result did not survive an FDR < 0.05. FEP patients, in comparison to GAP controls, showed greater cPLEs (B: 3.74, 95% CI: 1.37 to 6.12) as part of the main effect of group (*χ*^*2*^(2) = 10.56, *p* = 0.005). There was no main effect of AKT1. There was no overall interaction between AKT1 and group (*χ*^*2*^(2) = 5.69, *p* = 0.06).

**Table 2:** Adjusted mixed effect model predicting cannabis-induced psychotic-like experiences (cPLE) and euphoric experiences (CEE) from covariates, AKT1 genotype and the interaction between AKT1 and group (GAP controls (*n* = 48); YA cannabis users (*n* = 442) and patients (*n* = 87)). Each model contains a random effects parameter of “participant”.

	cPLEs			cEEs		
	B	95% CI	*p*	B	95% CI	*p*
cEE	**0.09**	**0.04 to 0.14**	**0.003**	–	–	–
cPLE	–	–	–	**0.24**	**0.11 to 0.36**	**<0.001**
Age	−0.13	−0.25 to -0.02	0.02	−0.20	−0.39 to -0.02	0.03
Sex	−0.36	−0.98 to 0.27	0.26	−0.53	−1.53 to 0.48	0.30
Ethnicity	−0.00	−0.07 to 0.07	0.74	0.00	−0.10 to 0.12	0.92
Age of first cannabis use	−0.12	−0.26 to 0.03	0.11	0.19	−0.04 to 0.43	0.10
Frequency of cannabis use	−0.14	−0.42 to 0.12	0.29	−0.36	−0.79 to 0.08	0.11
Years of cannabis use	0.04	−0.08 to 0.15	0.54	**0.28**	**0.10 to 0.46**	**0.003**
Group^a^						
YA Cannabis users	1.71	−0.54 to 3.98	0.13	0.56	−3.05 to 4.17	0.76
FEP Patients	**3.74**	**1.37 to 6.12**	**0.002**	0.83	−3.02 to 4.66	0.67
AKT1	2.02	−0.13 to 4.18	0.066	0.41	−3.05 to 3.87	0.81
group^a^AKT1^b^						
YA cannabis users × AKT1	−2.29	-4.57 to -0.00	0.05	−0.32	−3.98 to −3.34	0.86
FEP Patients × AKT1	−3.33	−6.09 to −0.57	0.02	−0.54	−4.98 to 3.89	0.81
Constant	10.88	7.67 to 14.01	<0.001	16.28	11.11 to 21.44	<0.001
N	578			578		
Wald χ^2^(12)	64.25 *p* < 0.001			36.62 *p* < 0.001		

^a^Reference category: GAP controls.

^b^Reference category: GAP controls with AKT1 homozygote TT genotype; multiple comparisons are corrected with a FDR of 0.05. Results in bold are significant.

In regards to cEEs, there was a positive association with cPLEs (B: 0.24, 95% CI: 0.12 to 0.37). This model also showed a negative association with age suggesting younger individuals were experiencing greater euphoric effects of cannabis (B: −0.2 95% CI:−0.39 to −0.02). There was a positive association with years of cannabis use (B: 0.28, 95% CI: 0.1 to 0.46) wherein those individuals with more years of cannabis use had experiencing greater cEEs. There was no main effect of AKT1 or group or interaction between AKT1 and group on cEEs.

#### COMT

In the model predicting cPLEs, apart from a positive association of cEEs with cPLEs (B: 0.1, 95% CI: 0.05 to 0.15), there were no other associations (Table 3). In regards to cEEs, there was a positive association with cPLEs (B: 0.24, 95% CI: 0.12 to 0.37). There was also a negative association with frequency of cannabis use, such that greater cannabis use was related to greater cEEs (B:−0.43, 95%CI: −0.85 to −0.02), and a positive association with years of cannabis use such that those with greater years experienced more cEEs (B: 0.17, 95% CI: 0.02 to 0.33).

**Table 3:** Adjusted mixed effect model predicting cannabis-induced psychotic-like experiences (cPLE) and euphoric experiences (CEE) from covariates, COMT genotype and the interaction between COMT and group (GAP controls (*n* = 66); YA cannabis users (*n* = 432) and patients (*n* = 94)). Each model contains a random effects parameter of “participant”.

	cPLEs			cEEs		
	B	95% CI	*p*	B	95% CI	*p*
cEE	**0.10**	**0.05 to 0.15**	**<0.001**	–	–	–
cPLE	–	–	–	**0.24**	**0.12 to 0.37**	**<0.001**
Age	−0.10	−0.19 to 0.00	0.06	−0.14	−0.30 to 0.02	0.08
Sex	−0.49	−1.10 to 0.13	0.13	−0.65	−1.64 to 0.35	0.20
Ethnicity	−0.01	−0.08 to 0.06	0.72	0.04	−0.07 to 0.15	0.50
Age of first cannabis use	−0.12	−0.26 to 0.01	0.07	0.09	−0.13 to 0.30	0.45
Frequency of cannabis use	−0.18	−0.44 to 0.09	0.19	−0.43	−0.85 to -0.02	0.04
Years of cannabis use	−0.01	−0.09 to 0.11	0.87	0.17	0.02 to 0.33	0.03
Group^a^						
YA Cannabis users	−0.13	−2.80 to 2.55	0.93	−0.99	−5.27 to 3.27	0.65
FEP Patients	1.47	−1.66 to 4.87	0.34	4.37	−0.84 to 9.58	0.10
COMT	−0.46	−2.90 to 1.98	0.71	−0.74	−4.65 to 3.16	0.70
group^a^COMT^b^						
YA cannabis users × COMT	0.70	−1.85 to 3.24	0.59	1.00	−3.08 to 5.06	0.64
FEP Patients × COMT	−0.14	−3.54 to 3.25	0.93	−4.62	−10.04 to 0.80	0.09
Constant	11.98	8.41 to 15.55	<0.001	18.52	12.81 to 24.24	<0.001
N	592			592		
Wald χ^2^(12)	64.25 *p* < 0.001			46.10 *p* < 0.001		

^a^Reference category: GAP controls.

^b^Reference category: GAP controls with homozygote COMT AA (MET/MET) genotype; multiple comparisons are corrected with a FDR of 0.05. Results in Bold are significant.

#### FAAH

Similar to the above models, there was a positive association between cEEs and cPLEs (B: 0.08, 95%CI: 0.03 to 0.13) in the model predicting cPLEs (Table 4). We also saw a negative association with age of first cannabis use, such that those who started using cannabis earlier reported greater cPLEs (B: −0.16, 95% CI: −0.29 to −0.03). There was a main effect of group (*χ*^*2*^(2) = 31.46, *p* < 0.001) as evidenced by lower cPLEs in the YA cannabis users than GAP controls (B: −1.76, 95% CI: −3.25 to −0.28; *p* = 0.02). There was no main effect of FAAH and there was no interaction between group and FAAH. In regards to the model predicting cEEs, there was a positive association between cPLE and cEE (B: 0.24, 95% CI: 0.11 to 0.37). There was a significant positive association between years of cannabis use and cEEs (B: 0.19, 95% CI: 0.04 to 0.35). There was no main effect of FAAH or group or interaction with FAAH.

**Table 4:** Adjusted mixed effect model predicting cannabis-induced psychotic-like experiences (cPLE) and euphoric experiences (CEE) from covariates, FAAH genotype and the interaction between FAAH and group (GAP controls (*n* = 61); YA cannabis users (*n* = 434) and patients (*n* = 95)). Each model contains a random effects parameter of “participant”.

	cPLEs			cEEs		
	B	95% CI	*p*	B	95% CI	*p*
cEE	**0.08**	**0.03 to 0.13**	**<0.001**	–	–	–
cPLE	–	–	–	**0.24**	**0.11 to 0.37**	**<0.001**
Age	−0.07	−0.17 to 0.03	0.15	−0.14	−0.30 to 0.01	0.06
Sex	−0.47	−1.08 to 0.15	0.14	−0.73	−1.71 to 0.29	0.13
Ethnicity	−0.02	−0.09 to 0.05	0.60	0.03	−0.09 to 0.14	0.57
Age of first cannabis use	**−0.16**	**−0.29 to −0.03**	**0.02**	0.14	−0.74 to 0.35	0.14
Frequency of cannabis use	−0.04	−0.28 to 0.21	0.77	−0.53	−0.94 to −0.12	0.15
Years of cannabis use	−0.00	−0.10 to 0.10	0.97	**0.19**	**0.04 to 0.35**	**0.01**
Group^a^						
YA Cannabis user	**−1.76**	**−3.25 to −0.28**	**0.02**	0.52	−2.22 to 3.26	0.71
FEP Patients	0.69	−0.86 to 2.24	0.38	−0.48	−2.96 to 2.00	0.70
FAAH	−0.70	−2.42 to 1.03	0.43	−0.06	−2.68 to 2.81	0.96
group^a^FAAH^b^						
YA cannabis users × FAAH	0.62	−1.24 to 2.47	0.51	−0.11	−3.06 to 2.84	0.94
FEP Patients × FAAH	0.75	−1.47 to 2.96	0.51	2.03	−1.49 to 5.54	0.25
Constant	13.30	10.53 to 16.07	<0.001	16.75	12.17 to 21.33	<0.001
N	590			590		
Wald χ^2^(12)	68.62, *p* < 0.001			43.85 *p* < 0.001		

^a^Reference category: GAP controls.

^b^Reference category: GAP controls with homozygote FAAH CC genotype; multiple comparisons are corrected with a FDR of 0.05. Results in bold are significant.

### Sensitivity analysis

In the above analyses, we have controlled for the confounding effects of ethnicity. However, for COMT and FAAH there are population-wide differences between the across the main (black and white Europeans) ethnic groups^62^, but this is not the case for AKT1^37^. Allele frequency by ethnicity reported as a percentage of the total sample can be found in supplementary table 7. Therefore, we replicated the above analysis twice. Once without individuals whose self-reported ethnicity was black African, black Caribbean and black Other (supplementary materials Tables 1–3 sensitivity analysis 1) and once only in those who self-reported being white European or white other (supplementary materials Table 4–6 sensitivity analysis 2). The findings can be seen in the supplementary materials. In short, the results remain very similar to the main analysis reported above but with less power due to decreases in sample size.

## Discussion

In this study, as hypothesised, we found that FEP patients experienced greater cPLEs than GAP controls and YA cannabis users, but YA cannabis users experienced the greatest cEEs, followed by FEP patients then GAP controls. This study did not find any association between the Val158Met polymorphism of the COMT gene with cPLEs or cEEs whilst accounting for demographics and cannabis use variables. Our findings add to the existing mixed findings that the meta-analysis by Vaessen et al.^31^, suggested are likely due to differences in study design regarding the control group. Our study, is the first to include two independent control groups, which reduces the likelihood of over-estimating the true effect which case-only studies do.

Our findings regarding AKT1 contrast with three previously published studies. In Morgan et al.^29^ the acute change in the psychotomimetic effect of smoked cannabis was modulated by the AKT1 genotype wherein the C allele was associated with greater intoxicated psychotomimetic symptoms. COMT had no effect. Di Forti et al.^37^, replicated the Van Winkel et al.^39^ case-control data, showing that CC genotype carriers of AKT1 with a history of cannabis use showed a two-fold increase in the ORs for a psychotic disorder, in comparison to TT carriers and an interaction with the genotype and frequency of use was found on case/control status. They found that among daily users, C carriers had a seven-fold increase in the odds of psychosis diagnosis.

A key difference between this study and Morgan et al.^29^ is the measure of cPLE. We used the CEQ, a retrospective measure of nine items in comparison to Morgan et al.^29^ who used the PSI (under acute cannabis exposure), which has better test-retest reliability than other scales designed to tap psychotic-like effects^63^. Further, Di Forti et al.^37^ and van Winkel et al.^39^ did not measure cPLE but OR for case-control status, which was diagnosed by a clinician.

Indeed, this is the first study to investigate the role of the FAAH rs324420 genotype in relation to cPLEs. Previous evidences have suggested a possible role of the eCB system in psychosis. Patients suffering from all stages of psychotic disorders have increased plasma and cerebrospinal fluid levels of anandamide, independent of antipsychotic treatment and current cannabis use^43,64^. However, previous population-wide and postmortem research on the FAAH rs324420 genotype, where the A allele leads to the 30% reduction in the functioning of the enzyme^47–49^ have not shown any associations with schizophrenia^52,53^. We did not find evidence that the FAAH rs324420 SNP was associated with either CEQ measure. Future research with case/control status and cannabis use is warranted based on research that suggests anandamide is also downregulated in chronic cannabis users^45^. We did not have evidence for the functional consequences of FAAH or the other SNPs. This is important because there may be neurobiological variation related to having FEP or regular cannabis use, which may modulate anandamide levels. Indeed, future studies should aim to externally validate the consequences of the SNPs examined. More work is needed to investigate role of the eCB system in schizophrenia and cannabis-induced experiences, as previous research has shown that the FAAH rs324420 SNP is involved in behavioural manifestations of cannabis addiction^59^. In these times of changing cannabis legislation, research highlighting the biological effects of cannabinoids is greatly needed in the face of concerns about unintended negative consequence of cannabis use.

The hypothesis that these specific genetic SNPs would be involved in the acute cannabis experience was based on previous research suggesting that these SNPs have a causal effect on the biological systems associated with development of psychosis. However, while genetic factors appear to play a role in the relationship between cannabis and psychosis, it is also clear that other environmental factors are critically important. In our statistical models, we found that an increase in one subscale is highly associated with an increase in the other subscale, suggesting cPLEs and cEEs are highly correlated. Additionally, we found differences between groups on cPLEs and cEEs. We also see strong effects of cannabis use itself; years of cannabis use predicted cEEs in both the AKT1 and FAAH models and age of first cannabis use predicted cPLEs in the FAAH model. This pattern of results has been found previously in both local^12^ and multinational samples^61^. Moreover, an interesting pattern of results emerges in the models in regards to age, years of cannabis use and cEEs. In all cEE models, there were negative associations with age, such that those who are younger, experienced greater euphoric effects of cannabis. There were positive associations with years of cannabis use, suggesting the more years of cannabis use, the greater the euphoric experiences. Overall, this may suggest that younger, more experienced cannabis users, regardless of group, may be most sensitive to the acute euphoric experiences of cannabis such as feeling happy, understanding the world better and being full of plans or ideas. While genetic factors may play some mediating role between cannabis and psychosis, the contribution of a range of environmental factors, such as population density^65^ and childhood trauma^66^ and their interaction with genotype is less understood.

Although in the present study we concentrated on three SNPs that have been previously been highlighted in research on the putative biological mechanisms associating cannabis and psychosis, data that arises from GWAS should guide future research. The fact that the SNPs investigated in this analysis have not been investigated and replicated as causal variants in schizophrenia GWAS limits the interpretation of these findings. Particularly, the expression of the neuronal acetylcholine receptor alpha-2 subunit CHRNA2 was found to be significant in the GWAS of cannabis use disorder^67^ and in the largest schizophrenia GWAS^68^. Additionally there may be a strong biological link between the expression of CHRNA2 and the gene that encodes the cannabinoid receptor type 1 (CNR1), which is based on the assessment of neuroanatomically precise, genome-wide maps of gene-expression correlations^69^.

### Strengths and limitations

The whole sample of this study was relatively large (*n* = 720), allowing exploration of if and how AKT1, COMT and FAAH interact with different participant group on cannabis-induced psychotic-like and euphoric experiences. Although the groups analyses indicated significant differences in demographics and cannabis-related variables between the FEP, GAP controls and YA. Therefore, the findings from the group comparisons should be interpreted cautiously. We chose the three SNPs in question based on a strong hypothesis-driven rationale and best genetic coverage, to guarantee a good quality and reliable genetic analysis. Indeed, this is why the CNR1 gene was not included as it did not have good coverage. Limitations of this study include the behavioural genetics approach, which utilises candidate genes that are typically common variants, and as such only have small effects, hampering our power to detect effects. The multi-ethnic nature of the sample may also be considered a limitation because COMT and FAAH variant frequencies show differences between the minor allele frequency estimates in African and European populations^62^. However, as well as controlling for ethnicity, we conducted sensitivity analyses, which did not significantly modify the results. Further, we did not investigate genotype in an additive manner but in a binary fashion in order to increase power. It should be noted that HWE was violated for the distribution of FAAH in GAP control participants only; however, this did not modify the overall HWE for FAAH. We assessed baseline group differences in age, gender, ethnicity and cannabis use variables (age of first use, frequency, and years of use) but other potential confounders, such as the type of cannabis used (THC:CBD ratio), grams per day or whether cannabis was smoked with tobacco, were not assessed. Recall bias may have reduced the reliability of the CEQ and the retrospective measure of the patterns of cannabis use. Since THC has an acutely amnestic effect, retrospective reporting of intoxicated experience is necessarily questionable. Retrospective reports would be influenced by other experiences such as the anxiogenic effects of the drug.

## Conclusions

Investigating the underlying mechanisms of the potential association between cannabis and risk for psychosis is crucial for the better understanding of the aetiology of psychotic disorders and for the development of prevention interventions. This study combined a well characterised large sample of FEP cases and controls with a naturalistic study of young adult recreational cannabis users. We found that FEP patients experienced greater cPLEs than GAP controls and YA cannabis users, but YA cannabis users experienced the greatest cEEs, followed by FEP cases and then controls. Whilst controlling for a range of confounders, including demographics and multiple indicators of cannabis use, there was no evidence that AKT1, COMT of FAAH influenced cannabis-induced psychotic-like or euphoric experiences. Further, there was no evidence for interactions between these SNPs and group, on cannabis-induced experiences. Future direction might focus on building genetic pathways scores based on eCB system relevant SNPs to further explore its role in shaping individual susceptibility to the psychotogenic effect of heavy cannabis use.

## Supplementary information

Supplementary materials
