# Supplementary material for: Do AKT1, COMT and FAAH influence reports of acute cannabis intoxication experiences in patients with first episode psychosis, controls and young adult cannabis users?
Source: Transl Psychiatry. 2020 May 12;10:143. doi: 10.1038/s41398-020-0823-9 (PMC7217850; doi:10.1038/s41398-020-0823-9)
Supplement: Supplementary file 1 — Supplementary materials [file 41398_2020_823_MOESM1_ESM.pdf]

SUPPLEMENTARY MATERIALS

Do AKT1, COMT and FAAH influence reports of acute cannabis intoxication experiences in patients with first episode psychosis, controls and young adult cannabis users?

Chandni Hindocha [1,2,3], Diego Quattrone[4, 5, 6], Tom Freeman[1,2,7], Robin M Murray[5,8], Valeria Mondelli[9], Gerome Breen[4,5,10], Charles Curtis[4,5,10] Celia Morgan[1,11], H Valerie Curran[1,3], Marta Di Forti[4, 5, 6]

Supplementary material 1: Unadjusted simple linear regression analyses between each variable and cPLEs and CEEs

cPLEs

Gender was negatively associated with cPLEs (B: -0.71, 95% CI: -1.283 to -0.128) such that males were more likely to experience cPLEs than females. Age of first cannabis use was negatively associated with cPLEs (B: -0.17, 95%CI: -0.26 to -0.08) where those who started cannabis younger showed greater symptoms. Frequency of cannabis use was positively associated with cPLEs (B:0.29, 95% CI: 0.07 to 0.51) with those using everyday experiencing more cPLES than those who used rarely. Finally group was associated with cPLEs (B:0.95, 95% CI: 0.48 to 1.42) with patients experiencing the most cPLEs.

Age was not associated with cPLEs (B:-0.41, 95% CI:-0.08 to 0.00), neither was ethnicity (B: -0.04, 95% CI: -0.11 to 0.03). Years of cannabis use was not associated with cPLEs (B:0.02, 95% CI: -0.03 to 0.07). AKT1 was not associated with cPLEs (B:0.01, 95% CI: -0.66 to 0.68), neither was COMT (B:0.41, 95% CI: -0.28 to 1.02) nor FAAH (B:0.30, 95% CI: -0.28 to 0.88).

cEEs

Age was negatively associated with cEEs (B: -0.16, 95% CI: -0.22 to -0.09), as was gender (B:-1.42, 95% CI: -2.35 to -0.49) such that males were more likely to experience cEEs than females. Age of first cannabis use was negatively associated with cEEs such that those who started using younger experienced greater cEEs (B:-0.27, 95% CI: -0.42 to -0.13). Frequency of cannabis use was associated with cEEs (B:-1.12, 95% CI: -1.47 to -0.77)

Ethnicity was not associated with cEEs (B: 0.03, 95% CI: -0.14 to 0.08), neither was years of cannabis use (B: -0.03, 95% CI: -0.11 to 0.06). There was no significant association between group and cEEs (B:0.44, 95% CI: -0.33 to 1.21) AKT1 was not significantly associated with cEEs (B:0.25, 95% CI: -0.80 to 1.30), neither was COMT (B:-0.536, 95% CI: -0.17 to 0.60) or FAAH (B:0.06, 95% CI:-0.88 to 1.00).

Sensitivity Analyses 1

Additional sensitivity analyses were conducted due to differences across racial groups. The tables below are a replication of the analysis found in the main text, but without " Black African", "Black Caribbean" and " Black Other".

Supplementary Table 1: Adjusted mixed effect model predicting cannabis induced psychotic like experiences (cPLE) and euphoric experiences (CEE) from covariates, AKT1 (rs2494732) genotype and the interaction between AKT1 and group (GAP controls (n=35); YA cannabis users (n=425) and patients (n=69)). Each model contains a random effects parameter of "participant"

	**cPLEs**			**cEEs**		
	B	95%CI	p	B	95%CI	p
**cEE**	0.07	0.02 to 0.13	0.009	-	-	-
**cPLE**	-	-	-	0.19	0.05 to 0.32	0.009
**Age**	-0.12	-0.25 to 0.00	0.05	-0.16	-0.36 to 0.04	0.11
**Sex**	-0.33	-0.99 to 0.33	0.98	-0.28	-1.35 to 0.79	0.61
**Ethnicity**	-0.007	-0.08 to 0.07	0.85	-0.01	-0.13 to 0.11	0.85
**Age of first cannabis use**	-0.18	-0.36 to -0.01	0.04	0.24	-0.04 to 0.52	0.09
**Frequency of cannabis use**	0.06	-0.22 to 0.33	0.68	-0.44	-0.88 to 0.00	0.05
**Years of cannabis use**	0.02	-0.11 to 0.15	0.72	**0.31**	**0.10 to 0.51**	**0.003**
**Group***						
YA cannabis users	-0.25	-2.95 to 2.45	0.86	4.42	0.09 to 8.75	0.05
FEP Patients	3.54	0.21 to 6.86	0.04	2.86	-2.52 to 8.21	0.30
**AKT1**	2.10	-0.70 to 4.90	0.14	0.94	-3.57 to 5.45	0.68
**Group*AKT1**$^{\Delta}$						
YA cannabis users x AKT1	-2.36	-5.25 to 0.53	0.11	-0.88	-5.50 to 3.79	0.72
FEP Patients X AKT1	-3.04	-6.76 to 0.69	0.11	-2.04	-8.05 to 3.97	0.51
**Constant**	13.17	9.12 to 17.27	0.00	11.89	5.16 to 18.63	0.00
**N**	509			509		
**Wald χ^2(12)**	50.92 p <0.001			42.37 p<0.001		

*Notes: *reference category: GAP controls; $^{\Delta}$ reference category: GAP controls with AKT1 homozygote TT genotype; multiple comparisons are corrected with a FDR of 0.05.*

Supplementary Table 2: Adjusted mixed effect model predicting cannabis induced psychotic like experiences (cPLE) and euphoric experiences (CEE) from covariates, COMT (Val158Met (rs4680)), genotype and the interaction between COMT and group (GAP controls (n=51); YA cannabis users (n=415) and patients (n=55)). Each model contains a random effects parameter of "participant"

	**cPLEs**			**cEEs**		
	B	95%CI	p	B	95%CI	p
**cEE**	**0.08**	**0.03 to 0.13**	**0.004**	-	-	-
**cPLE**	-	-	-	**0.20**	**0.07 to 0.34**	**0.004**
**Age**	-0.09	-0.20 to 0.02	0.09	-0.11	-0.28 to 0.06	0.21
**Sex**	-0.41	-1.07 to 0.24	0.21	-0.42	-1.48 to 0.64	0.44
**Ethnicity**	-0.02	-0.09 to 0.05	0.60	0.03	-0.09 to 0.15	0.66
**Age of first cannabis use**	-0.17	-0.33 to -0.01	0.04	0.11	-0.96 to -0.10	0.02
**Frequency of cannabis use**	0.00	-0.27 to 0.26	0.98	-0.53	-0.85 to -0.02	0.04
**Years of cannabis use**	-0.01	-0.11 to 0.10	0.91	0.19	0.01 to 0.36	0.03
**Group***						
YA Cannabis users	-1.94	-4.58 to 0.70	0.15	2.20	-2.10 to 6.49	0.32
FEP Patients	0.32	-3.56 to 4.21	0.87	5.54	-0.748 to 11.83	0.08
**COMT**	-0.30	-2.80 to 2.21	0.82	-0.63	-4.70 to 3.44	0.76
**Group*COMT**$^{\Delta}$						
YA Cannabis users x COMT	0.41	-2.20 to 3.02	0.59	1.07	-3.16 to 5.30	0.64
FEP Patients X COMT	0.97	-3.01 to 5.03	0.64	-5.03	-11.61 to 1.55	0.13
**Constant**	14.05	10.00 to 18.09	0.00	15.22	8.50 to 21.95	0.00
**N**	521			521		
**Wald $\chi^2(12)$**	44.74 p<0.001			49.57 p<0.001		

*Notes: *reference category: GAP controls; $^{\Delta}$ reference category: GAP controls with homozygote COMT AA (MET/MET) genotype; multiple comparisons are corrected with a FDR of 0.05.*

Supplementary Table 3: Adjusted mixed effect model predicting cannabis induced psychotic like experiences (cPLE) and euphoric experiences (CEE) from covariates, FAAH (rs324420) genotype and the interaction between FAAH and group (GAP controls (n=46); YA cannabis users (n=417) and patients (n=62)). Each model contains a random effects parameter of "participant"

	**cPLEs**			**cEEs**		
	B	95%CI	p	B	95%CI	p
**cEE**	0.08	0.02 to 0.13	0.005	-	-	-
**cPLE**	-	-	-	**0.19**	**0.06 to 0.34**	**0.005**
**Age**	-0.07	-0.16 to 0.03	0.19	-0.10	-0.26 to 0.06	0.21
**Sex**	-0.42	-1.06 to 0.22	0.20	-0.64	-1.68 to 0.40	0.22
**Ethnicity**	-0.03	-0.10 to 0.05	0.47	0.01	-0.11 to 0.13	0.85
**Age of first cannabis use**	-0.18	-0.34 to - 0.03	0.02	0.17	-0.08 to 0.43	0.18
**Frequency of cannabis use**	-0.03	-0.29 to 0.23	0.81	**-0.57**	**-0.99 to -0.16**	**0.007**
**Years of cannabis use**	-0.02	-0.12 to 0.09	0.77	0.18	0.01 to 0.35	0.04
**Group***						
YA Cannabis users	-1.80	-3.37 to -0.14	0.03	3.02	0.40 to 5.65	0.02
FEP Patients	0.09	-1.61 to 1.80	0.91	0.07	-2.69 to 2.84	0.95
**FAAH**	-0.54	-2.50 to 1.42	0.59	-0.39	-3.57 to 2.79	0.81
**Group*FAAH**$^{\Delta}$						
YA cannabis users x FAAH	0.39	-1.69 to 2.46	0.71	0.32	-3.04 to 3.68	0.85
FEP Patients X FAAH	1.82	-0.83 to 4.48	0.18	2.61	-1.70 to 6.91	0.24
**Constant**	13.74	10.46 to 17.03	0.00	13.92	8.38 to 19.45	0.00
**N**	525			525		
**Wald χ^2(12)**	40.54, p<0.001			49.29, p<0.001		

Notes: *reference category: GAP controls; $^{\Delta}$ reference category: GAP controls with homozygote FAAH CC genotype; *multiple comparisons are corrected with a FDR of 0.05.*

Sensitivity Analyses 2

Additional sensitivity analyses were conducted due to differences across racial groups. The tables below are a replication of the analysis found in the main text, but only in the white European group which represent the largest ethnic subgroup in the data. It should be noted that this reduces the sample size a great deal, and therefore such results should be considered as preliminary.

Supplementary Table 4: Adjusted mixed effect model predicting cannabis induced psychotic like experiences (cPLE) and euphoric experiences (CEE) from covariates, AKT1 (rs2494732) genotype and the interaction between AKT1 and group (GAP controls (n=28); YA cannabis users (n=292) and patients (n=32)). Each model contains a random effects parameter of "participant"

	**cPLEs**			**cEEs**		
	B	95%CI	p	B	95%CI	p
**cEE**	0.06	-0.00 to 0.13	0.06	-	-	-
**cPLE**	-	-	-	0.15	-0.01 to 0.32	0.06
**Age**	-0.10	-0.24 to 0.03	0.14	-0.19	-0.40 to 0.02	0.08
**Sex**	-0.41	-1.21 to 0.39	0.98	-0.29	-1.53 to 0.95	0.64
**Age of first cannabis use**	-0.24	-0.47to -0.02	0.03	0.21	-0.14 to 0.56	0.24
**Frequency of cannabis use**	0.05	-0.28 to 0.39	0.75	-0.42	-0.94 to 0.10	0.12
**Years of cannabis use**	0.01	-0.14 to 0.16	0.88	0.29	0.06 to 0.52	0.01
**Group***						
YA cannabis users	-1.13	-4.45 to 2.20	0.51	6.59	1.48 to 11.70	0.01
FEP Patients	2.61	-1.18 to 6.40	0.18	5.59	-0.27 to 11.50	0.06
**AKT1**	0.92	-2.44 to 4.28	0.59	3.32	-1.85 to 8.50	0.21
**Group*AKT1**$^{\Delta}$						
YA cannabis users x AKT1	-1.38	-4.85 to 2.09	0.44	-2.63	-8.00 to 2.75	0.34
FEP Patients X AKT1	-1.91	-6.23 to 2.41	0.39	-3.82	-10.51 to 2.86	0.26
**Constant**	14.78	9.61 to 19.95	P<0.001	10.76	2.48 to 19.04	0.02
**N**	352			352		
**Wald χ^2(12)**	33.46 p=0.004			47.96 p<0.001		

*Notes: *reference category: GAP controls; $^{\Delta}$ reference category: GAP controls with AKT1 homozygote TT genotype; multiple comparisons are corrected with a FDR of 0.05.*

Supplementary Table 5: Adjusted mixed effect model predicting cannabis induced psychotic like experiences (cPLE) and euphoric experiences (CEE) from covariates, COMT (Val158Met (rs4680)), genotype and the interaction between COMT and group GAP controls (n=42); YA cannabis users (n=285) and patients (n=37)). Each model contains a random effects parameter of "participant"

	cPLEs			cEEs		
	B	95%CI	p	B	95%CI	p
**cEE**	0.06	-0.00 to 0.13	0.07	-	-	-
**cPLE**	-	-	-	0.15	-0.01 to 0.31	0.07
**Age**	-0.08	-0.19 to 0.04	0.21	-0.13	-0.31 to 0.06	0.17
**Sex**	-0.58	-1.35 to -0.20	0.15	-0.38	-1.60 to 0.84	0.54
**Age of first cannabis use**	-0.21	-0.42 to -0.01	0.04	0.00	-0.32 to 0.33	0.99
**Frequency of cannabis use**	-0.03	-0.36 to 0.30	0.86	-0.50	-1.00 to 0.01	0.06
**Years of cannabis use**	-0.01	-0.13 to 0.11	0.85	0.15	-0.03 to 0.33	0.11
**Group***						
YA Cannabis users	-1.85	-4.63 to 0.92	0.19	1.21	-3.14 to 5.57	0.59
FEP Patients	0.63	-3.34 to 4.60	0.76	5.25	-0.95 to 11.45	0.10
**COMT**	-0.34	-2.95 to 2.27	0.80	-1.46	-5.55 to 2.63	0.48
**Group*COMT**$^{\Delta}$						
YA Cannabis users x COMT	0.44	-2.31 to 3.19	0.75	2.50	-1.80 to 6.81	0.25
FEP Patients X COMT	0.79	-3.47 to 5.04	0.72	-3.36	-10.01 to 3.30	0.32
**Constant**	14.51	9.86 to 19.16	<0.001	18.57	11.16 to 25.99	<0.001
**N**	364			364		
**Wald $\chi^2(12)$**	28.63 p=0.003			42.94 p<0.001		

*Notes: *reference category: GAP controls; $^{\Delta}$ reference category: GAP controls with homozygote COMT AA (MET/MET) genotype; multiple comparisons are corrected with a FDR of 0.05.*

Supplementary Table 6: Adjusted mixed effect model predicting cannabis induced psychotic like experiences (cPLE) and euphoric experiences (CEE) from covariates, FAAH (rs324420) genotype and the interaction between FAAH and group (n=28); YA cannabis users (n=287) and patients (n=44)). Each model contains a random effects parameter of "participant"

	**cPLEs**			**cEEs**		
	B	95%CI	p	B	95%CI	p
**cEE**	0.06	-0.01 to 0.12	0.07	-	-	-
**cPLE**	-	-	-	0.15	-0.01 to 0.31	0.07
**Age**	-0.04	-0.15 to 0.06	0.41	-0.12	-0.29 to 0.05	0.16
**Sex**	-0.64	-1.40 to 0.13	0.10	-0.70	-1.91 to 0.51	0.26
**Age of first cannabis use**	-0.22	-0.40 to -0.03	0.03	0.12	-0.18 to 0.41	0.43
**Frequency of cannabis use**	-0.05	-0.36 to 0.26	0.76	-0.53	-1.02 to-0.04	0.04
**Years of cannabis use**	-0.02	-0.13 to 0.10	0.79	0.15	-0.03 to 0.33	0.09
**Group***						
YA Cannabis users	-1.55	-3.34 to 0.25	0.09	3.60	0.77 to 6.43	0.01
FEP Patients	-0.30	-2.17 to 1.57	0.75	2.72	-0.23 to 5.67	0.07
**FAAH**	-0.55	-2.74 to 1.63	0.62	0.66	-2.80 to 4.11	0.71
**Group*FAAH**$^{\Delta}$						
YA cannabis users x FAAH	0.15	-2.20 to 2.49	0.90	-0.59	-4.29 to 3.11	0.75
FEP Patients X FAAH	3.45	0.37 to 6.54	0.03*	-0.97	-5.88 to 3.94	0.70
**Constant**	13.92	10.09 to 17.76	<0.001	15.25	8.97 to 21.53	<0.001
**N**	369			369		
**Wald χ^2(12)**	40.54, p<0.001			35.30, p=0.0002		

Notes: *reference category: GAP controls; $^{\Delta}$ reference category: GAP controls with homozygote FAAH CC genotype; *multiple comparisons are corrected with a FDR of 0.05.*

Supplementary Fig. 1

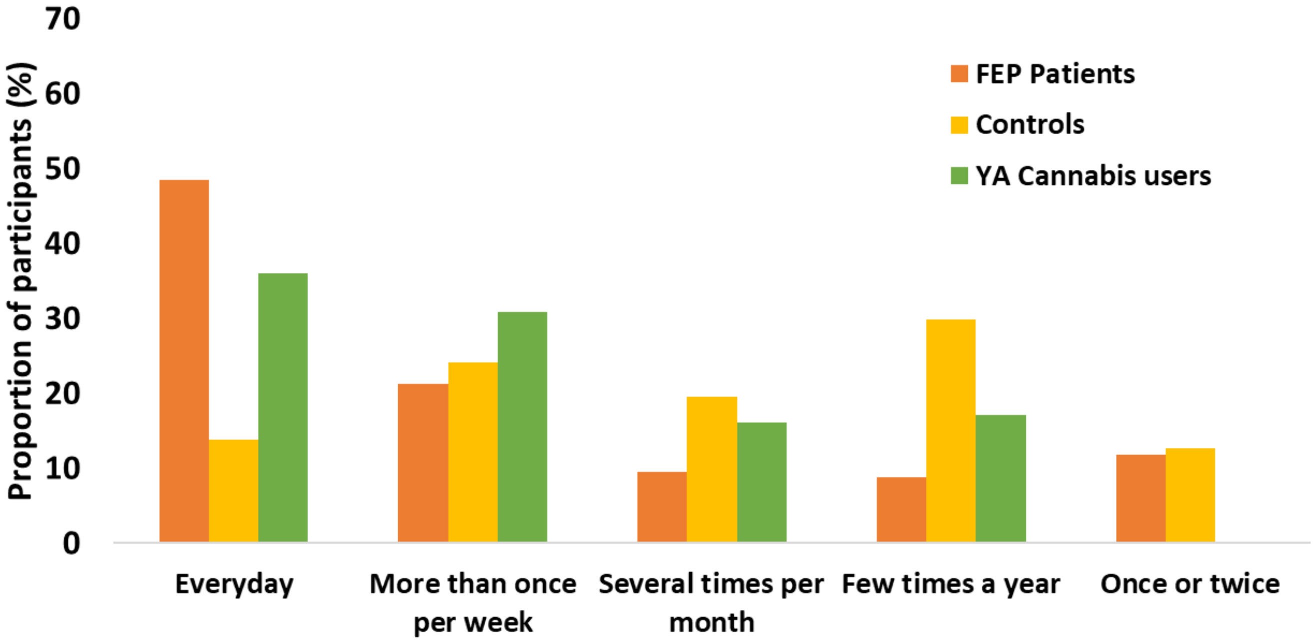

Suppl Fig. 1: The proportion of individuals (%) within each of the three groups (FEP patients controls and young adult (YA) cannabis users) who reported their frequency of cannabis use. The sampling strategy for YA cannabis users precluded users who had only used once or twice.

Supplementary Table 7: Allele frequency by self-reported ethnicity reported as % of the total sample

	AKT1		COMT		FAAH	
	TT	CC or CT	AA	AG or GG	CC	AA or AC
WHITE BRITISH	14.66	35.17	12.80	36.89	32.47	18.07
WHITE OTHER	3.00	8.35	1.98	10.21	8.12	4.13
MIXED	0.63	5.84	0.91	5.64	3.83	2.60
INDIAN	1.74	4.10	1.37	4.27	3.83	1.84
PAKISTANI	0.32	0.95	0.15	0.91	1.07	0.15
BANGLADESHI	0.16	0.32	0	0.45	0.15	0.31
OTHER ASIAN	1.10	5.84	1.98	5.03	4.44	2.76
BLACK CARIBBEAN	1.58	5.36	0.91	6.10	2.60	3.52
BLACK AFRICAN	0.95	4.26	0.76	4.42	2.60	2.45
BLACK OTHER	0.16	0	0.15	0	0.15	0
CHINESE	0	2.05	0.15	1.83	1.53	0.46
OTHER	0.79	2.68	0.61	2.44	1.68	1.22
N	634		656		635	
